# Measuring the Emergence of Specific Abilities in Young Children with Autism Spectrum Disorders: The Example of Early Hyperlexic Traits

**DOI:** 10.3390/brainsci11060692

**Published:** 2021-05-25

**Authors:** Stefania Solazzo, Nada Kojovic, François Robain, Marie Schaer

**Affiliations:** Department of Psychiatry, Faculty of Medicine, University of Geneva, 1206 Geneva, Switzerland; Nada.Kojovic@unige.ch (N.K.); Francois.Robain@unige.ch (F.R.); Marie.Schaer@unige.ch (M.S.)

**Keywords:** restricted interests, letters and numbers, special skills, early literacy, hyperlexia

## Abstract

The presence of a restricted interest in written materials, including an early ability to name and recognize letters and numbers, is regularly reported in preschoolers with autism spectrum disorders (ASDs). There is, however, scarce information on this early ability akin to emerging hyperlexic traits in preschoolers with ASD younger than 3 years old. Here, we defined a measure of early naming and recognition of letters and numbers in 155 preschoolers with ASD using a sliding window approach combined with a 90th percentile threshold criterion, and subsequently compared the profiles of children with ASD with and without early hyperlexic traits. Using this measure, we found that 9% of children with ASD showed early hyperlexic traits. The early ability to name and recognize letters and numbers was associated with a higher level of restricted and repetitive behaviors yet more social-oriented behaviors at baseline and with better expressive and written communication at baseline and one year later. This study contributes to a better definition of the profile of children with ASD with an early ability in letters and numbers akin to emerging hyperlexic traits, a skill that is associated with promising social strengths and language abilities in this subgroup of children.

## 1. Introduction

Autism spectrum disorders (ASDs) are a group of neurodevelopmental disorders characterized by impaired social communication and interaction coupled with restricted and repetitive behaviors [1]. Restricted and repetitive behaviors (RRBs) include a variety of behaviors that can be divided into two categories labeled as “lower level” and “higher level” RRBs [1,2]. “Lower level” RRBs are predominant in younger children and include sensory seeking or aversion related to hypo- or hyper-reactivity to sensory stimuli and repetitive, stereotyped movements. “Higher level” RRBs include restricted and repetitive interests, ritualization of behaviors, insistence on maintaining routines and these tend to occur in older children. Although they may change over the course of a child’s development, these two types of behaviors are not mutually exclusive and can coincide. Research on RRBs focuses primarily on their detrimental effects as they can be difficult to manage [3,4] or stigmatizing [5] for the family and also have a major impact on the child’s learning [6]. However, RRBs do not systematically interfere with the daily functioning of children with ASD [7]. Among individuals with ASD, some develop a strong restricted interest in a particular domain, resulting in a subsequent specific skill such as absolute pitch discrimination [8], calendar calculation [9], exceptional drawing [10,11] and hyperlexia [12,13,14,15]. Such strengths arising from “higher level” RRBs are poorly described, particularly when they emerge in children with ASD who do not present a higher functioning profile, and hyperlexia is no exception. Yet, their investigation is crucial considering the potential leverage in learning that they can represent, especially for children with early learning disabilities.

Hyperlexia is defined as an early word reading skill in the context of a neurodevelopmental disorder, along with an interest in written material, that is acquired without any explicit teaching and is superior to language comprehension and general cognitive level [15,16]. Hyperlexia is therefore described as a “superability” to read words rather than a disability in comprehension [17] and is hence part of the special skills found in ASD. While it is also observed in populations with developmental delay [12], Down syndrome [18], Turner syndrome [19] and attention deficit hyperactivity disorder [20], the frequency of hyperlexia is higher in ASD compared to other neurodevelopmental disorders [12,16]. Indeed, hyperlexia in ASD ranges from 6 to 20% and occurs predominantly in boys [16]. The high prevalence of hyperlexia reported in ASD makes it an especially relevant population in which to investigate the early emergence of this special skill. 

The presence of an early and unusually intense specific interest in written material, including letters and numbers, is one of the most consistently reported RRBs in ASD [21,22,23,24]. When this strong interest does not remain purely visual but appears in conjunction with a particular ability to recognize and name letters and numbers, it may predispose children to develop hyperlexia [16]. Moreover, it has been suggested that in children with ASD and hyperlexia, a strong interest in reading results in an increase in time and practice devoted to this ability, thus leading to the emergence of advanced reading skills [25]. The timeline of the emergence of the interest in written material underlying hyperlexia is poorly documented in the literature but emerging evidence suggests its presence from a very early age. For example, in a naturalistic play design, children with ASD between 20 and 69 months old explored more literacy-related objects than typically developing (TD) children [26]. In addition, an eye-tracking study showed that, even though most of them could not read, children with ASD aged from 2.8 to 9 years old were attracted to a caption in a video depicting a girl talking, while TD children did not visually explore this information [27]. Similarly, enhanced visual search of letters in 9-month-old infants at high risk for ASD predicted increased levels of symptoms at 15 and 24 months old [28]. 

The impact that this early interest in literacy-related content exerts on the downstream developmental processes remains elusive. As for many RRBs, this early interest in literacy could be considered invasive as it may prevent children from engaging in other activities, hinder their learning and become a source of concerns for parents [29]. Nevertheless, this interest may prove beneficial when learning to decode words and favor subsequent academic learning through written material [16]. Indeed, in alphabetic writing systems, alphabetic decoding is the very first step to acquire the principle that letters represent sounds [30]. Letter identification appears to be a prerequisite to this principle and is a key skill in inducing subsequent word decoding. Regarding the emergence of letter naming, it has been shown that children with ASD between 4 and 7 years old tend to name more letters than their TD peers at the same age [31,32], but this gap disappears around first grade [31]. Furthermore, children with ASD and hyperlexia have better letter naming skills than their ASD as well as TD peers [33].

Unlike most ASD co-occurring disorders during infancy and adolescence [34,35], hyperlexia is often considered a positive prognosis marker in ASD. Indeed, studies report an increased cognitive level, better expressive and receptive language skills, improved adaptive behavior and reduced autism symptom severity in school-aged children with hyperlexia [16,36,37,38]. Conversely, fewer studies did not observe higher receptive and expressive language skills nor improved outcomes in children with hyperlexia regarding their expressive communication and adaptive functioning [12,33], suggesting that the profiles associated with hyperlexia are not yet clearly established. Although the reading skills of children with hyperlexia ultimately become similar to those of children typically developing at around 10 years of age [29,39], this advanced skill is a valuable strength in ASD, a disorder primarily known to have an adverse impact on academic learning, due to its association with impairments in language, executive functions or cognition [40,41,42,43]. Accordingly, it is beneficial to identify the premises of this special ability, which is scarcely explored at this early stage in the literature, as it can be a valuable asset in the context of intervention and can support learning in areas of weakness in children with ASD [29,44].

To our knowledge, there is scarce information on the emergence of hyperlexic traits in preschoolers with ASD younger than 3 years old. In this study, we were interested in exploring the advanced ability in letters and numbers akin to early emergence of hyperlexic premises in young children with ASD. The aims of the study were (1) to measure the early recognition and naming of letters and numbers and word reading in preschoolers with ASD, (2) to identify the profile of children with ASD presenting early hyperlexic traits regarding their ASD symptoms, cognitive level and adaptive functioning compared to their ASD and TD peers and (3) to explore their developmental trajectory. We leveraged a large sample of 155 preschoolers with ASD, with a subgroup of 129 that was followed-up after a one-year interval. A smaller group of 30 TD children matched for age was used for comparison purposes. To capture early manifestation of emerging hyperlexic traits, we constrained our analysis to children between 2 and 5 years old and defined a novel metric to quantify early hyperlexic traits. We investigated the potential differences in autistic symptoms and developmental profile of children with ASD in relation to their level of early hyperlexic traits. We hypothesized that children with ASD with emerging hyperlexic traits would show better expressive language skills than their ASD peers and equally low comprehension skills at baseline, according to the hyperlexia definition [15,16]. We also hypothesized that higher early hyperlexic traits would be associated with a greater progression in expressive language skills over one year.

## 2. Materials and Methods

### 2.1. Participants

All participants included in the current study were assessed as part of the Geneva Autism Cohort [45] between 2012 and 2020. This project was approved by the University of Geneva Institutional Review Board and all parents gave their written consent for their child’s participation in the study. The children with ASD were recruited through clinical centers specialized in developmental disorders and parent associations in the Geneva area, whereas their TD peers were recruited through announcements. In the present study, all children with ASD received a diagnosis based on the DSM-5 criteria [1] using standardized diagnostic assessments and the presence of any autistic traits was excluded in all TD children. All children were assessed with the Autism Diagnosis Observation Schedule Generic (ADOS-G; n = 48) [46] or the Autism Diagnosis Observation Schedule, second edition (ADOS-2; n = 137) [47]. Moreover, the TD participants were screened for the presence of any neurodevelopmental disorder. Children included in the present study were all assessed with the Psycho-Educational Profile, third edition (PEP-3) [48]. The final sample (see Table 1) was composed of 185 preschoolers aged between 2 and 5 years old, comprising 155 children with ASD (*ASD*; 2.95 ± 0.72 years old; 20 females) and 30 TD children (*TD*; 2.87 ± 0.79 years old; 9 females). As detailed below, children with ASD were subsequently divided into two groups according to the presence (*ASD+eHPL*; n = 14; 2.87 ± 0.54 years old; 1 female) or absence (*ASD-eHPL*; n = 141; 2.96 ± 0.73 years old; 19 females) of early emerging hyperlexic traits measured by a *hyperlexic trait score* that was derived from the PEP-3 (see definition of the measure below). 

Of the 155 children with ASD, a total of 129 children had a follow-up assessment one year later. Among these children, 117 belonged to the *ASD-eHPL* group and 12 belonged to the *ASD+eHPL* group based on their results at baseline. In a secondary step of the analyses, this longitudinal subsample was used to explore developmental change over time. The 12 children from the *ASD+eHPL* group and the 117 children from the *ASD-eHPL* group did not differ regarding their age (*F*(1,127) = 0.100, *p* = 0.752) or sex (χ^2^(1, n = 129) = 0.140, *p* = 0.709).

### 2.2. Measures

We established the *hyperlexic trait score* based on a selection of PEP-3 items described above to measure the advanced ability in letter and number naming, recognition and word reading, akin to early hyperlexic trait manifestation. The *hyperlexic trait score* aimed to identify this early literacy ability akin to hyperlexic traits in young children with ASD solely, as hyperlexia does not apply to TD [15,16]. The 8 following items were selected from the PEP-3 and composed the *hyperlexic trait score*: Identify nine letters correctly, Name nine letters correctly, Read numbers from one to ten correctly, Read three words, Read a short sentence correctly, Read a passage with less than three errors, Read a passage and answer two comprehension questions correctly, Read a sentence and follow the instruction (items 88, 89, 91–96). Each item was rated on a three-level scale (Failing = 0 point; Emerging = 1 point; Passing = 2 points) following standardized scoring procedures validated for the PEP-3. The *hyperlexic trait score* corresponded to the sum of the raw scores of each item and therefore ranged from a minimum of 0 to a maximum of 16 points.

**Figure 1:**
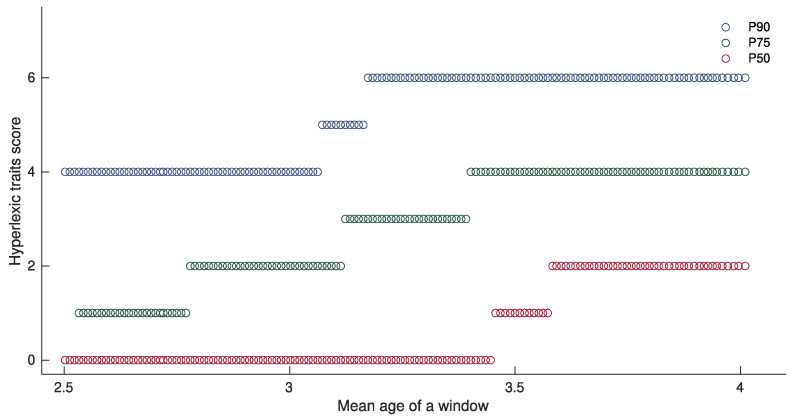
Distribution of the *hyperlexic trait score* across partially overlapping age windows, each comprising 100 unique visits, according to 90th, 75th and 50th percentile threshold criteria, respectively, represented in blue, green and red.

Considering that hyperlexia is described as the early emergence of reading, a skill considered extraordinary for its age of onset, it was important to identify the highest abilities in letters and numbers measured by the *hyperlexic trait score* by age group. Furthermore, the skills measured by the *hyperlexic trait score* evolve with age, so a fixed threshold was not the best option. In order to take these factors into account, we sought to determine a strict and age-appropriate cut-off for our measure. To accurately account for age in the development of early skills in letters and numbers, we applied a sliding window strategy [49] on all available data using a custom MATLAB R2019b (The MathWorks, Inc., Natick, MA, USA) script. For the 155 children with ASD included in this study, we had follow-up data with at least two *hyperlexic trait scores* for 91 of them, and three *hyperlexic trait scores* for 24 of them, summing up to 270 observations obtained, respectively, at one- or two-year intervals. We used all of these 270 individual scores to extract age-dependent percentiles. A window comprising 100 visits was slid across the 270 observations ordered according to age, advancing one observation at a time. The script excluded the repeated observations of the same child, ensuring that no child contributed more than one observation in a given window. This procedure yielded 147 age windows (see Figure 1) with the first window corresponding to a mean age of 2.50 (SD = 0.28), and the last to a mean age of 4.01 (SD = 0.34). For each window, we applied a 90th percentile criteria to define the *hyperlexic trait score* value characterizing the best performing 10% of the participants of the selected window that would serve as a threshold to define the presence or absence of early hyperlexic traits. The distribution of the age-dependent cut-off values is illustrated in Figure 1 and is also detailed in the Results section. This procedure allowed us to identify two subgroups among the ASD participants: a first group of children with ASD and an advanced ability in letters and numbers, akin to early hyperlexic traits (*ASD+eHPL*), and a second group of children with ASD without any early hyperlexic traits (*ASD-eHPL*). 

The symptoms of autism were assessed using the ADOS-G or ADOS-2 [46,47]. An appropriate ADOS module was chosen with respect to the age and language level of each child, following standard procedures. The ADOS-calibrated severity scores were used to assess the total severity of symptoms score (Total), restricted and repetitive behaviors severity score (RRB) and social affect severity score (SA) on a ten-point scale, with the exception of the RRB scale which did not include scores 2 to 4 [50,51]. To get a fine-grained insight on which of the individual symptoms relates the most to the emerging hyperlexia-like traits, we used the raw scores of the 27 ADOS items that are shared across the used modules (Modules Toddler, 1, 2 and 3). These items were rated on a four-point scale (ranging from 0 to 3), with a higher score indicating more abnormal behaviors.

Regarding the cognitive measures, we used the Mullen Scales of Early Learning (MSEL) developmental quotients (DQs) for four subdomains: Visual Reception, Fine Motor, Receptive Language, Expressive Language [52]. In addition, we used the Visual–Motor Imitation subdomain of the PEP-3 to obtain a measure of imitation skills. Given that the norms for the Visual–Motor Imitation scale are only available up to 3.5 years old, we chose to analyze its raw scores in order to avoid any ceiling effect.

To assess the adaptive functioning, we used the Vineland Adaptive Behavior Scales, second edition (VABS-II) [53] a semi-structured interview exploring the domains of Communication, Daily living skills, Socialization and Motor skills. We looked more precisely into the subscales of Communication, namely the Expressive, Receptive and Written subscales. Standardized and v-scale scores are provided for, respectively, each domain and subscale; raw scores are provided for both. As the Written subscale is only valid from the age of 3, we decided to analyze its raw scores, as it was often administered for clinical interest or when the children presented a known interest in letters and numbers despite their young age.

For the longitudinal part of the study, we calculated change scores to measure the evolution over one year for all the variables described above. Each change score corresponded to the subtraction between the score one year later and the baseline score. 

### 2.3. Analyses Strategy

Preliminary steps leading to the creation of the measure allowing us to identify early abilities in letter and number recognition and naming (*hyperlexic trait score*) were performed using a custom MATLAB R2019b (The MathWorks, Inc., Natick, MA, USA) script. We then compared our three groups on their symptoms, cognitive abilities, imitation skills and adaptive functioning at baseline. All analyses were carried using IBM SPSS Statistics (IBM Corp. Released 2019. IBM SPSS Statistics for Macintosh, Version 26.0. Armonk, NY, USA, IBM Corp.) and graphs were produced with GraphPad PRISM 8.0 (GraphPad Prism version 9.1.0 for Macintosh, GraphPad Software, San Diego, CA, USA, www.graphpad.com) or using a custom MATLAB R2019b script. To compare sample demographics, we conducted Student’s *t*-tests and chi-square tests (*ASD* vs. *TD*). Regarding the cross-sectional analyses, we used Mann–Whitney tests for two-group comparisons (*ASD-eHPL* vs. *ASD+eHPL*) and we used Kruskal–Wallis tests with post hoc comparisons using Bonferroni corrections when comparing three groups (*ASD-eHPL* vs. *ASD+eHPL* vs. *TD*). The U statistic of the test (*U*) and median and interquartile range (*IQR*) as well as the standard score (*z*), p-value (*p*) and effect size (*r*) values are reported for all tests in the Results section or Supplementary Table S1. We also performed Spearman correlations (*r_s_*) between the *hyperlexic trait score* and clinical assessments within the ASD sample exclusively (*ASD-eHPL* and *ASD+eHPL)*, for which we applied the Bonferroni correction for multiple correlations, dividing the level of *p* of 0.05 by the number of constructs measured on an identical scale. We thus used the thresholds of *p* < 0.016 (0.05/3) for the ADOS severity scores and *p* < 0.002 (0.05/27) for the ADOS individual items. Regarding the MSEL domains, results were considered significant at *p* < 0.013 (0.05/4). For the VABS-II domains, we used *p* < 0.013 (0.05/4) and for its Communication subscales, we used *p* < 0.016 (0.05/3). Concerning the longitudinal analyses, we performed Spearman correlations between the *hyperlexic trait score* at baseline and the difference scores in clinical assessments as well as between the *hyperlexic trait score* at baseline clinical measures one year later. We also applied the Bonferroni correction for multiple correlations and used the same aforementioned thresholds of significance.

Apart from the ADOS severity scores and Visual–Motor Imitation skills measure, which were available for the entire sample, there were some missing data. A total of 148 observations (n = 125 *ASD* and 23 *TD*) were available for the MSEL, which was introduced at a later stage in our research protocol. For the VABS-II, we collected 181 observations (n = 152 *ASD* and 29 *TD*) for the domains and subdomains assessed, except for the Written subscale, administered from age 3 onwards, for which we had 85 observations (n = 75 *ASD* and 10 *TD*). Regarding the individual items of the ADOS, the number of participants varied as item administration depended on the age and language level of the children, therefore causing differences in sample sizes. For the longitudinal part of the study, we collected 128 ADOS observations (n = 116 *ASD-eHPL* and 12 *ASD+eHPL*), 106 MSEL observations (n = 92 *ASD-eHPL* and 12 *ASD+eHPL*), 121 observations of visual–motor imitation skills provided by the PEP-3 (n = 110 *ASD-eHPL* and 11 *ASD+eHPL*) and 124 observations for the VABS-II (n = 114 *ASD-eHPL* and 10 *ASD+eHPL*), including 60 observations for the Written Communication subscale (n = 52 *ASD-eHPL* and 8 *ASD+eHPL*).

## 3. Results

### 3.1. Description of the ASD Sample Derived from the Hyperlexic Trait Score

The distribution of the *hyperlexic trait score* values included in the 90th percentile across age is illustrated in Figure 1. The following age-dependent thresholds on the *hyperlexic trait score* were used to decide whether each child’s score corresponded to early hyperlexic traits or not. (1) For children under 3.06 years of age, the *hyperlexic trait score* threshold was a minimum of 4. (2) For children under 3.16 years of age, the *hyperlexic trait score* threshold was a minimum of 5. (3) For the children under 4.01 years of age, the *hyperlexic trait score* threshold was a minimum of 6.

Using the *hyperlexic trait score* 90th percentile thresholds, we identified two subgroups amid the 155 children with ASD from our sample: 14 presented early letter and number skills (*ASD+eHPL*) and the remaining 141 did not show special competences in this area (*ASD-eHPL*). Most of the *ASD+eHPL* children matched the first threshold (*hyperlexic trait score* = 4; n = 11; 2.63 ± 0.29 years old; 1 female) and the rest matched the third threshold (*hyperlexic trait score* = 6; n = 3; 3.74 ± 0.15 years old). The *ASD+eHPL* (2.87 ± 0.54 years old; one female), *ASD-eHPL* (2.96 ± 0.73 years old; 19 females) and *TD* (2.87 ± 0.79 years old; nine females) groups did not differ in age (*F*(2,182) = 0.256, *p* = 0.775) or sex (*χ^2^*(2, n = 185) = 5.994, *p* = 0.052).

As the mean age of the first window used to identify the 90th percentile of the distribution was 2.50 years old, and the mean of the last window was 4.01 years old, the measure was unable to categorize children younger than 2.50 and older than 4.01 years old if their *hyperlexic trait score* was, respectively, between 1 and 3 and greater than 6 (see Figure 1). However, in our sample, no children older than 4.01 years old had a score higher than 6, and only eight children younger than 2.50 years old had a score higher than 0 (scores of 1 or 2).

As a reminder, we were interested in the distribution of the *hyperlexic trait score* only for the children with ASD, however, we would like to point out that none of the *TD* children in our study exceeded the above thresholds for early hyperlexic traits. Indeed, while the *ASD+eHPL* group had a higher *hyperlexic trait score* than *ASD-eHPL* and *TD* groups, no difference was found between the latter two groups (*U_ASD-eHPL/ASD+eHPL_* = −88.175, *p* < 0.001, *r* = −0.535; *U_ASD-eHPL/TD_* = −2.215, *p* =1.000, *r* = −0.019; *U_ASD+eHPL/TD_* = 85.960, *p* < 0.001, *r* = 0.451; see Supplementary Table S1). Moreover, no children in our sample showed early word reading as the most observed skills corresponded to the first three items included in the *hyperlexic trait score* (Identify nine letters correctly, Name nine letters correctly, Read numbers from one to ten correctly).

To ensure that the *hyperlexic trait score* was not simply detecting the children with better verbal abilities of our sample, we compared the level of *hyperlexic trait score* between the 14 children from the *ASD+eHPL* group and 14 children from the *ASD-eHPL* group (*ASD-eHPL_14*), both showing equivalent MSEL Verbal DQs which include Receptive and Expressive Language scales. The MSEL Verbal DQs of the 14 children in the *ASD+eHPL* group were manually matched to 14 children from the *ASD-eHPL* group. In other words, for each child in the *ASD+eHPL* group, we manually selected a child from the *ASD-eHPL* group with the closest MSEL Verbal DQ to form the *ASD-eHPL_14* group. The results confirmed that the subsample of 14 *ASD-eHPL* children had a significantly lower *hyperlexic trait score* than the *ASD+eHPL* group (*Mdn_ASD-eHPL_14_* = 0, *IQR_ASD-eHPL_14_* = 3; *Mdn_ASD+eHPL_* = 6, *IQR_ASD+eHPL_* = 2; *U* = 24, *z* = −3.525, *p* < 0.001, *r* = −0.126) despite the same level of receptive and expressive language skills (*Mdn_ASD-eHPL_14_* = 57.099, *IQR_ASD-eHPL_14_* = 33.603; *Mdn_ASD+eHPL_* = 57.313, *IQR_ASD+eHPL_* = 42.163; *U* = 90, *z* = −0.368, *p* = 0.713, *r* = −0.013).

### 3.2. Cross-Sectional Results

#### 3.2.1. Group Differences

**Figure 2:**
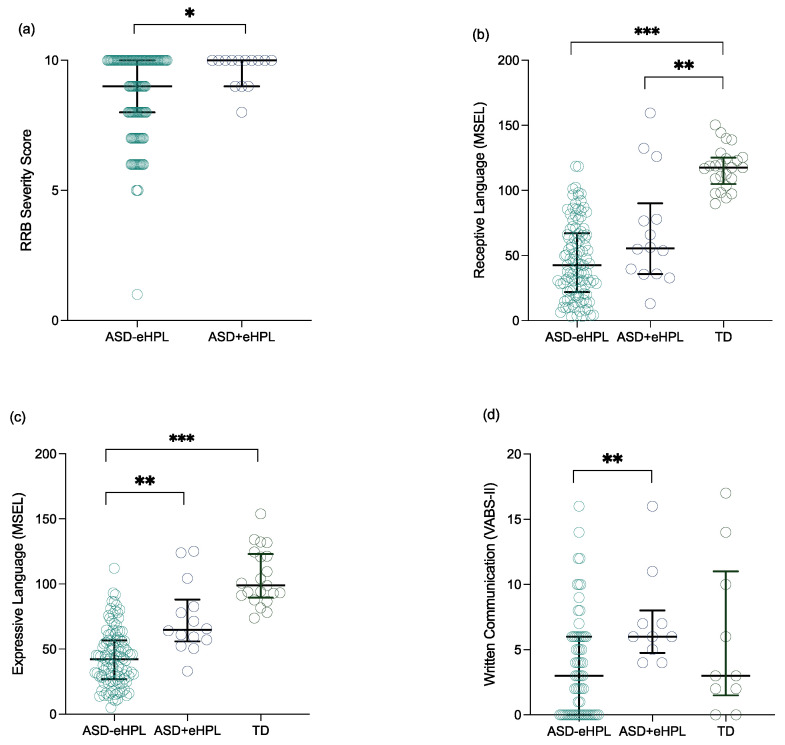
Group differences regarding autism symptoms and language skills. (**a**) Difference between the *ASD-eHPL* and *ASD+eHPL* groups on their RRB severity scores. (**b**) Differences between the *ASD-eHPL*, *ASD+eHPL* and *TD* groups in their receptive language skills (MSEL). (**c**) Differences between the *ASD-eHPL*, *ASD+eHPL* and *TD* groups on their expressive language skills (MSEL). (**d**) Differences between the *ASD-eHPL*, *ASD+eHPL* and *TD* groups in their written communication skills (VABS-II). * *p* < 0.05; ** *p* < 0.01; *** *p* < 0.001.

We first compared the symptom profiles of the two subgroups of children with ASD (Figure 2, Supplementary Table S1). The results showed higher RRB severity scores in the *ASD+eHPL* group compared to their *ASD-eHPL* peers (*U* = 661, *p* = 0.029, *r* = −0.175; see Figure 2a). No group differences were found in SA (*U* = 828, *p* = 0.316, *r* = −0.080) nor in total severity scores (*U* = 941, *p* = 0.771, *r* = −0.023).

We then compared the developmental skills and adaptive functioning scores between our two groups of children with ASD and the TD children (Figure 2, Supplementary Table S1). Children with *ASD+eHPL* showed better expressive language skills with the MSEL than children with *ASD-eHPL* but were equal to *TD* children, whereas *ASD-eHPL* presented lower skills compared to the *TD* group (*U_ASD-eHPL/ASD+eHPL_* = −42.848, *p* = 0.001, *r* = −0.292; *U_ASD-eHPL/TD_* = −72.900, *p* < 0.001, *r* = −0.603; *U_ASD+eHPL/TD_* = −30.052, *p* = 0.117, *r* = −0.170; see Figure 2c). Regarding the receptive language skills assessed by the MSEL, the *ASD-eHPL* and *ASD+eHPL* groups had similar scores but both were lower compared to the *TD* children (*U_ASD-eHPL/ASD+eHPL_* = −22.861, *p* = 0.175, *r* = −0.013; *U_ASD-eHPL/TD_* = −70.566, *p* < 0.001, *r* = −0.049; *U_ASD+eHPL/TD_* = −47.705, *p* = 0.003, *r* = −0.022; see Figure 2b). The *ASD+eHPL* and *ASD-eHPL* groups did not differ significantly from each other but both had lower performances than the *TD* group in their visual reception skills (*U_ASD-eHPL/ASD+eHPL_* = −11.086, *p* = 1.000, *r* = −0.006; *U_ASD-eHPL/TD_* = −64.586, *p* < 0.001, *r* = −0.436; *U_ASD+eHPL/TD_* = −53.500, *p* = 0.001, *r* = −0.025) and fine motor skills (*U_ASD-eHPL/ASD+eHPL_* = −15.911, *p* = 0.572, *r* = −0.108; *U_ASD-eHPL/TD_* = −64.663, *p* < 0.001, *r* = −0.541; *U_ASD+eHPL/TD_* = −48.752, *p* = 0.002, *r* = −0.276) as assessed by the MSEL. Similarly, the *ASD+eHPL* and *ASD-eHPL* groups showed equivalent scores on the Visual–Motor Imitation scale of the PEP-3 which were both lower than the *TD* children’s scores (*U_ASD-eHPL/ASD+eHPL_* = −19.864, *p* = 0.554, *r* = −0.097; *U_ASD-eHPL/TD_* = −68.076, *p* < 0.001, *r* = −0.466; *U_ASD+eHPL/TD_* = −48.212, *p* = 0.016, *r* = −0.205).

Regarding the VABS-II parents’ reports, children from the *ASD+eHPL* group showed similar communication skills compared to their *ASD-eHPL* peers and both were lower than the *TD* group (*U_ASD-eHPL/ASD+eHPL_* = −31.418, *p* = 0.116, *r* = −0.059; *U_ASD-eHPL/TD_* = −83.106, *p* < 0.001, *r* = −0.578; *U_ASD+eHPL/TD_* = −51.688, *p* = 0.009, *r* = −0.220). More precisely, the *ASD+eHPL* and *ASD-eHPL* scores were both lower than the *TD* group scores concerning both the Receptive (*U_ASD-eHPL/ASD+eHPL_* = −15.208, *p* = 0.944, *r* = −0.075; *U_ASD-eHPL/TD_* = −80.899, *p* < 0.001, *r* = −0.565; *U_ASD+eHPL/TD_* = −65.691, *p* < 0.001, *r* = −0.281) and Expressive (*U_ASD-eHPL/ASD+eHPL_* = −34.561, *p* = 0.066, *r* = −0.170; *U_ASD-eHPL/TD_* = −89.309, *p* < 0.001, *r* = −0.624; *U_ASD+eHPL/TD_* = −54.748, *p* = 0.005, *r* = 0.234) subdomains of the Communication scale and no difference was observed between them. However, we identified a significant difference between the two groups of children with ASD regarding the Written Communication subscale, where children with *ASD+eHPL* presented higher skills in this area. The written communication skills of the *ASD+eHPL* group did not differ significantly from those of the *TD* group, which in turn did not differ from those of the *ASD-eHPL* group (*U_ASD-eHPL/ASD+eHPL_* = −23.288, *p* = 0.014, *r* = −0.031; *U_ASD-eHPL/TD_* = −7.638, *p* = 1.000, *r* = −0.100; *U_ASD+eHPL/TD_* = 15.650, *p* = 0.449, *r* = 0.156; see Figure 2d). As for the other VABS-II domains, the *ASD+eHPL* and *ASD-eHPL* groups showed similar performances to each other but were inferior to *TD* children regarding their daily living skills (*U_ASD-eHPL/ASD+eHPL_* = −0.240, *p* = 1.000, *r* = −0.001; *U_ASD-eHPL/TD_* = −75.328, *p* < 0.001, *r* = −0.524; *U_ASD+eHPL/TD_* = −75.088, *p* < 0.001, *r* = 0.319), socialization skills (*U_ASD-eHPL/ASD+eHPL_* = −6.897, *p* = 1.000, *r* = −0.034; *U_ASD-eHPL/TD_* = −87.004, *p* < 0.001, *r* = −0.605; *U_ASD+eHPL/TD_* = −80.107, *p* < 0.001, *r* = 0.341) or motor skills (*U_ASD-eHPL/ASD+eHPL_* = −12.139, *p* = 1.000, *r* = −0.059; *U_ASD-eHPL/TD_* = −52.078, *p* < 0.001, *r* = −0.363; *U_ASD+eHPL/TD_* = −39.939, *p* = 0.066, *r* = 0.170).

#### 3.2.2. Correlations between the *Hyperlexic Trait Score* and Clinical Assessments at Baseline

**Figure 3:**
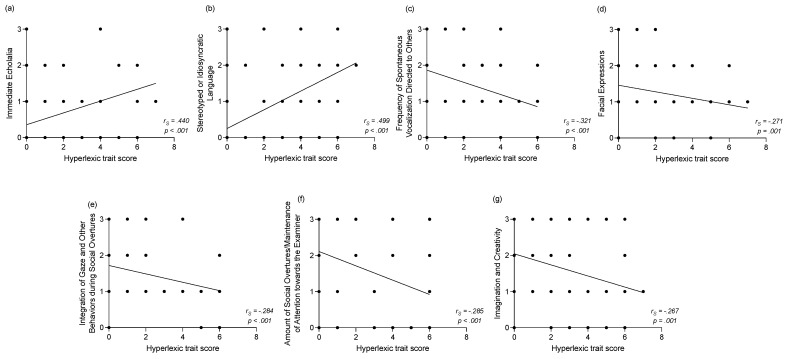
Correlations between the *hyperlexic trait score* and raw scores on individual ADOS items at baseline for the entire *ASD* group (comprising *ASD+eHPL* and *ASD-eHPL* groups). (**a**) Correlation between the *hyperlexic trait score* and immediate echolalia. (**b**) Correlation between the *hyperlexic trait score* and stereotyped or idiosyncratic language. (**c**) Correlation between the *hyperlexic trait score* and the frequency of spontaneous vocalization directed to others. (**d**) Correlation between the *hyperlexic trait score* and facial expressions. (**e**) Correlation between the *hyperlexic trait score* and integration of gaze and other behaviors during social overtures. (**f**) Correlation between the *hyperlexic trait score* and amount of social overtures/maintenance of attention towards the examiner. (**g**) Correlation between the *hyperlexic trait score* and imagination and creativity.

For children with ASD only, we explored the relationship between the *hyperlexic trait score* and clinical measures (diagnosis, developmental and adaptive functioning assessments) at baseline. In terms of ASD symptoms, we did not find any correlation between the *hyperlexic trait score* and SA (*r_s_* = −0.149, *p* = 0.064), RRB (*r_s_* = 0.109, *p* = 0.178) or Total (*r_s_* = −0.093, *p* = 0.251) scores of the ADOS. However, we observed several significant correlations between the *hyperlexic trait score* and individual ADOS raw scores at baseline (Figure 3). A higher *hyperlexic trait score* was associated with the presence of more immediate echolalia (*r_s_* = 0.440, *p* < 0.001, see Figure 3a) and stereotyped or idiosyncratic language (*r_s_* = 0.499, *p* < 0.001, see Figure 3b). In contrast, children with a higher *hyperlexic trait score* presented increased frequency of spontaneous vocalizations directed to others (*r_s_* = −0.321, *p* < 0.001, see Figure 3c) as well as more diversified and directed facial expressions (*r_s_* = −0.271, *p* < 0.001, see Figure 3d). They also showed more integration of gaze and other behaviors during social overtures (*r_s_* = −0.284, *p* < 0.001, see Figure 3e), presented an increased amount of social overtures/maintenance of attention towards the examiner (*r_s_* = −0.285, *p* < 0.001, see Figure 3f) and played with more imagination and creativity (*r_s_* = −0.267, *p* = 0.001, see Figure 3g). All other correlations with the ADOS individual items were not significant.

**Figure 4:**
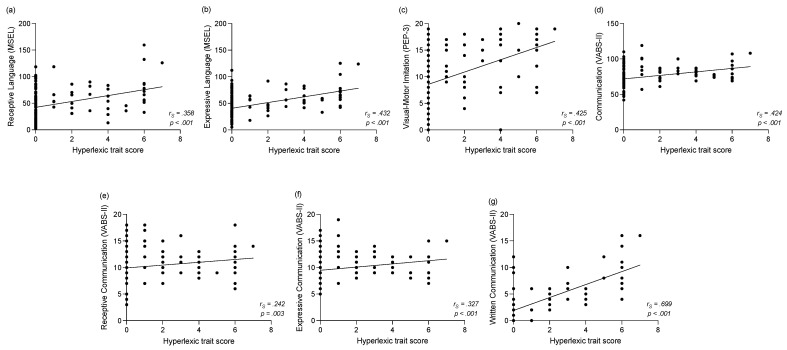
Correlations between the *hyperlexic trait score* and the developmental and adaptive assessments at baseline for the entire *ASD* group (comprising *ASD+eHPL* and *ASD-eHPL* groups). (**a**) Correlation between the *hyperlexic trait score* and receptive language skills (MSEL). (**b**) Correlation between the *hyperlexic trait score* and expressive language skills (MSEL). (**c**) Correlation between the *hyperlexic trait score* and visual–motor imitation skills (PEP-3). (**d**) Correlation between the *hyperlexic trait score* and communication skills (VABS-II). (**e**) Correlation between the *hyperlexic trait score* and receptive communication skills (VABS-II). (**f**) Correlation between the *hyperlexic trait score* and expressive communication skills (VABS-II). (**g**) Correlation between the *hyperlexic trait score* and written communication skills (VABS-II).

Regarding developmental skills at baseline, we identified significant relationships between the *hyperlexic trait score* and the language as well as imitation skills (Figure 4). Children with higher *hyperlexic trait score* showed better expressive (*r_s_* = 0.432, *p* < 0.001, see Figure 4b) and receptive (*r_s_* = 0.358, *p* < 0.001, see Figure 4a) language skills on the MSEL. However, the *hyperlexic trait score* did not significantly correlate with the visual reception (*r_s_* = 0.206, *p* = 0.021) or the fine motor skills (*r_s_* = 0.209, *p* = 0.019) of the MSEL after applying the correction for multiple correlations. Furthermore, a higher *hyperlexic trait score* was correlated to better visual–motor imitation skills in the PEP-3 (*r_s_* = 0.425, *p* < 0.001, see Figure 4c).

Regarding the parents’ reports on the VABS-II, a higher *hyperlexic trait score* was correlated to better overall communication scores (*r_s_* = 0.424, *p* < 0.001, see Figure 4d). More precisely, higher skills in receptive (*r_s_* = 0.242, *p* = 0.003, see Figure 4e), expressive (*r_s_* = 0.327, *p* < 0.001, see Figure 4f) and written communication (*r_s_* = 0.699, *p* < 0.001, see Figure 4g) were associated with a higher *hyperlexic trait score*. On the other hand, no significant correlation was found between the *hyperlexic trait score* and the daily living skills (*r_s_* = 0.077, *p* = 0.349), socialization skills (*r_s_* = 0.112, *p* = 0.170) and motor skills (*r_s_* = 0.016, *p* = 0.844).

### 3.3. Longitudinal Results

#### 3.3.1. Correlations between the *Hyperlexic Traits Score* and Change over Time

We did not identify any significant correlation between the change scores of the autism symptoms, developmental or adaptive measures over one year and the *hyperlexic trait score* in our longitudinal sample.

#### 3.3.2. Correlations between the *Hyperlexic Traits Score* and Clinical Assessments One Year Later

**Figure 5:**
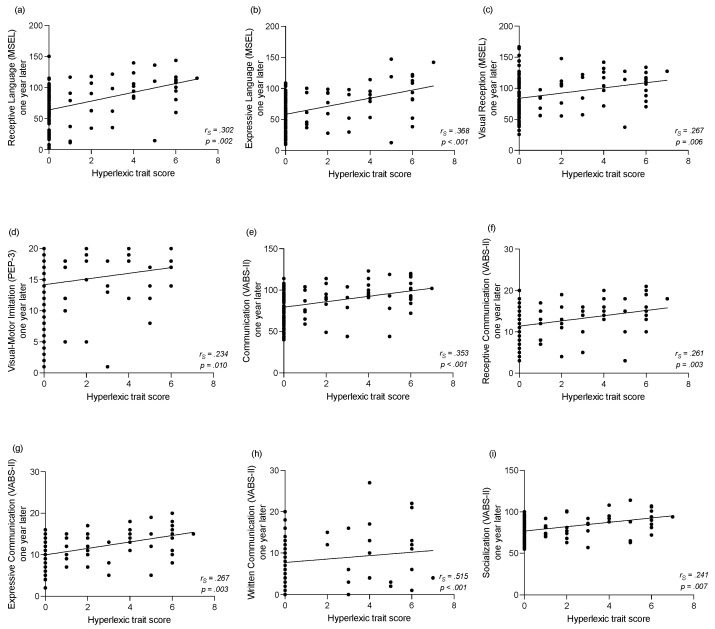
Correlations between the *hyperlexic trait score* at baseline and the developmental and adaptive assessments one year later for the longitudinal *ASD* group (comprising *ASD+eHPL* and *ASD-eHPL* groups). (**a**) Correlation between the *hyperlexic trait score* and receptive language skills one year later (MSEL). (**b**) Correlation between the *hyperlexic trait score* and expressive language skills one year later (MSEL). (**c**) Correlation between the *hyperlexic trait score* and visual reception skills one year later (MSEL). (**d**) Correlation between the *hyperlexic trait score* and visual–motor imitation skills one year later (PEP-3). (**e**) Correlation between the *hyperlexic trait score* and communication skills one year later (VABS-II). (**f**) Correlation between the *hyperlexic trait score* and receptive communication skills one year later (VABS-II). (**g**) Correlation between the *hyperlexic trait score* and expressive communication skills one year later (VABS-II). (**h**) Correlation between the *hyperlexic trait score* and written communication skills one year later (VABS-II). (**i**) Correlation between the *hyperlexic trait score* and socialization skills one year later (VABS-II).

We also explored the relationships between the *hyperlexic trait score* at baseline and clinical measures (diagnosis, developmental and adaptive functioning assessments) one year later (Figure 5). In terms of symptoms, we did not find any significant correlation between the *hyperlexic trait score* and SA (*r_s_* = 0.010, *p* = 0.909), RRB (*r_s_* = −0.022, *p* = 0.802) and Total (*r_s_* = −0.008, *p* = 0.929) scores or the individual items of the ADOS.

Significant correlations with developmental measures observed at baseline were still significant with measures one year apart. Regarding the MSEL, children with a higher *hyperlexic trait score* at baseline showed better receptive (*r_s_* = 0.302, *p* = 0.002, see Figure 5a) and expressive (*r_s_* = 0.368, *p* < 0.001, see Figure 5b) language skills one year later. Moreover, a higher *hyperlexic trait score* at baseline was also correlated with higher visual reception abilities one year later (*r_s_* = 0.267, *p* = 0.006, see Figure 5d) but not with motor skills (*r_s_* = 0.181, *p* = 0.064). Furthermore, a higher *hyperlexic trait score* at baseline was correlated to better visual–motor imitation skills (*r_s_* = 0.234, *p* = 0.010; see Figure 5c) in the PEP-3 one year later.

Based on the VABS-II parental reports, a higher *hyperlexic trait score* at baseline was associated with better overall communication (*r_s_* = 0.353, *p* < 0.001, see Figure 5e) as well as better receptive (*r_s_* = 0.261, *p* = 0.003, see Figure 5f), expressive (*r_s_* = 0.267, *p* = 0.003, see Figure 5g) and written (*r_s_* = 0.515, *p* < 0.001, see Figure 5h) communication skills one year later. Furthermore, a higher *hyperlexic trait score* at baseline was associated with more socialization skills (*r_s_* = 0.241, *p* = 0.007, see Figure 5i) after one year while no significant correlation was found with the daily living skills (*r_s_* = 0.219, *p* = 0.025), after applying the Bonferroni correction, or the motor skills (*r_s_* = 0.167, *p* = 0.064).

## 4. Discussion

In this study, we aimed to examine the developmental profiles of a subgroup of children with ASD who showed early abilities in recognition and naming of letters and numbers, akin to emerging hyperlexic traits. In that context, we used eight selected items from the PEP-3 to define a custom measure of early hyperlexic traits in children aged 2 to 5 years old, the *hyperlexic trait score*. In our cohort of 155 preschoolers with ASD, we identified a subsample of 14 children (9%, *ASD+eHPL*) who showed advanced abilities regarding letters and numbers although none of them presented the criteria for hyperlexia. These children with early hyperlexic traits were mostly younger than 3 and their profile was associated with more restricted and repetitive behaviors (RRB), stereotyped language and immediate echolalia. Nonetheless, our study revealed some primarily language-based advantages in the *ASD+eHPL* children over their peers with ASD (*ASD-eHPL*), using standardized developmental tests. Increased hyperlexic traits were also associated with more other-directed behaviors in social interaction and imagination in the ADOS as well as better imitation skills in the PEP-3. The *ASD+eHPL* group had a higher *hyperlexic trait score* than the age-matched *TD* group, confirming that early literacy-related interest and ability occurs primarily in ASD compared to typical development [31,32].

Regarding the symptom profile of our sample, the *ASD+eHPL* group showed higher RRB severity scores than the *ASD-eHPL* group. Several authors suggested that the presence of detail-focused RRBs such as an interest in written content, coupled with local processing of information, may be the cause of particular talents in ASD [54,55]. Thus, when more cognitive resources and time are allocated to the interest at hand, it may give rise to a particular area of expertise [54]. Looking more specifically into the individual symptoms comprising the ADOS RRB domain, our analyses revealed a positive correlation between the *hyperlexic trait score* and stereotyped or idiosyncratic language. Considering that this symptom tends to be more prevalent in children with ASD with higher cognitive abilities [56], its presence may not be as deleterious as “lower level” RRBs and could be promising regarding the cognitive profile of our *ASD+eHPL* group. Interestingly, no correlation was found between the *hyperlexic trait score* and “lower level” RRBs such as sensory interests or repetitive behaviors, indicating that the emergence of hyperlexic traits is more related to stereotypical aspects of language. An increased presence of emerging hyperlexic features in children with ASD was also associated with more immediate echolalia in our sample. This language particularity is often observed in both ASD and hyperlexia [57,58,59]. Nevertheless, immediate echolalia is described as functional in language development, and intervention processes should not target its elimination [60]. Indeed, in typical language acquisition, a substantial amount of learning occurs through repetition of another’s speech [61]. Although it is not always communicative and may persist over time in ASD, immediate echolalia remains indicative of language emergence.

No significant differences were observed for social affect (SA) and total severity scores between the *ASD+eHPL* and *ASD-eHPL* groups. However, at the individual symptom level, we found negative correlations between symptoms related to communication and social interaction in the ADOS and the *hyperlexic trait score.* Children with higher early literacy-related skills tended to direct their vocalizations and facial expressions more frequently, integrate gaze with gestures and vocalizations more often during social interactions and show more interaction initiation with the examiner during ADOS activities. The ability to promptly direct and coordinate behaviors towards others in social contexts is crucial for a fluid social interaction. However, the use of such communication tools and their combination are impaired in ASD [62,63]. Although the *hyperlexic trait score* did not correlate with global SA severity scores, the fact that some complex socially oriented behaviors appear to be preserved in the *ASD+eHPL* group is promising for their social functioning. Indeed, the combination of eye contact, facial expressions and vocalizations represents an important foundation which gradually develops during infancy and contributes to successful social exchanges [64]. In our sample, a higher *hyperlexic trait score* was also correlated to increased imagination and creativity scores from the ADOS. The development of imaginative play during childhood is also considerably involved in social development. The progressive acquisition of pretend play skills, which is subtended by basic functional play, is an important developmental milestone. However, its development may be delayed and impacted by RRBs in ASD [7,65,66]. Nevertheless, in our sample, the presence of a special interest in letters and numbers did not appear to impact play as it was linked to more imagination. Consistent with the results regarding the socio-communicative items of the ADOS, we found a positive correlation between the *hyperlexic trait score* at baseline and socialization skills, which include play skills and quality of social interaction, as reported by parents in the VABS-II after a one-year interval. This finding again supports the fact that the presence of early hyperlexic traits is related to more preserved social skills. Nevertheless, some confounding factors should be considered. Indeed, when controlling for expressive language skills using the Expressive Language scale of the MSEL, the correlations between the *hyperlexic trait score* and the aforementioned socially oriented behaviors are no longer significant, while the correlations with stereotyped or idiosyncratic language and immediate echolalia remain significant. Similarly, when controlling for the non-verbal cognition skills using the Visual Reception scale of the MSEL, only the correlations involving frequency of spontaneous vocalization directed to others, stereotyped or idiosyncratic language and immediate echolalia remain significant. However, when controlling for the ADOS SA severity scores, all correlations remain significant. It is therefore necessary to take into account the influence of language and non-verbal cognition on these results as it prevents us from drawing definite conclusions about the link between early hyperlexic traits and more socially directed behaviors in our sample.

Children’s advanced skills in naming and recognizing letters and numbers were also associated with differences in their expressive, receptive and written language skills compared to the rest of the sample. As we hypothesized, children with ASD with emerging hyperlexic traits showed better expressive language skills than their ASD peers and equally low comprehension skills. Regarding the expressive language measured with the MSEL, the *ASD+eHPL* group showed better expressive language than their *ASD-eHPL* peers and were similar to *TD* children. In line with our results differentiating the *ASD+eHPL* group from the *ASD-eHPL* group, it has been shown that better letter naming skills between 4 and 5 years old were related to better oral language skills [67]. In addition, this difference is supported by positive correlations between the *hyperlexic trait score* and expressive language assessed by the MSEL and VABS-II both at baseline and one year later. Conversely, the similar expressive language skills between *ASD+eHPL* and *TD* children was an unexpected result. Indeed, language is often affected in ASD [41,68] and yet the *ASD+eHPL* children showed age-appropriate skills. As the children in our sample were less than 3 years old on average, these findings do not imply that the gap will not widen as they grow older, despite their early literacy-related skills. Indeed, it has recently been shown that hyperlexic children with ASD between the ages of 3 and 6 had lower oral language skills than their *TD* peers [33]. Additionally, contrary to our hypothesis, the longitudinal analyses did not show any association between emerging hyperlexic traits and greater progress in expressive skills over one year, suggesting that the language advantage at baseline was not permanent in our sample. Nonetheless, our findings remain positive and encouraging for further language development in this subgroup of children with ASD.

The receptive language level measured with the MSEL was equivalent between children in *ASD+eHPL* and *ASD-eHPL* groups but lower than the one in *TD* children. This is consistent with the literature highlighting specific language patterns in autism, where the delay in comprehension seems to be more pronounced than the delay in expressive language [69,70]. Nevertheless, the *hyperlexic trait score* correlated with language comprehension levels assessed by the MSEL and VABS-II in our sample of children with ASD both at baseline and one year later. This suggests that the higher the letter and number skills of children with ASD, the better their comprehension. This is promising for subsequent language development as better language comprehension is related to better expressive language outcome [71].

The last group difference observed in our study was related to the VABS-II Written Communication subscale and revealed better skills in the *ASD+eHPL* and *TD* groups compared to the *ASD-eHPL* group. Consistent with these results, we also identified a strong correlation between the *hyperlexic trait score* and written communication among children with ASD at baseline and one year later. These results are not surprising considering that the Written Communication subscale items most frequently observed in the *ASD+eHPL* group included skills comparable to those measured by the *hyperlexic trait score*, such as letter recognition to varying degrees (e.g., recognition of only 10 letters or all letters in upper and/or lower case). Recently, it was shown that children with ASD between the ages of 4 and 6 tended to have more age-appropriate scores on the VABS-II Written Communication subscale than on the Receptive and Expressive Communication scales [72]. As many of the children in our sample were under 3 years of age, we were only able to collect raw scores on the Communication scale, not allowing us to make such comparisons based on developmental age. Nevertheless, in line with these last findings, children in the *ASD+eHPL* group showed written communication levels comparable to *TD* children, which is not the case for receptive and expressive communication assessed by the VABS-II, as they were both lower than *TD*. This could be explained by the fact that written communication skills are related to visual information processing, which is often described as an area of relative strength in the ASD profile, especially when compared to language skills [73]. Similarly, the Visual Reception scale assessed by the MSEL is strongly underpinned by reasoning based on visual information perception and processing. In our study, it is promising to observe that the higher presence of emerging hyperlexic traits was positively related to the visual reception skills one year later. Higher scores on visual reception can indeed reflect enhanced perceptual functioning and visual pattern recognition [74,75,76,77], which is highly relevant regarding the interest in written material such as letters and numbers.

Finally, better visual–motor imitation skills were also associated with an increased *hyperlexic trait score* in our sample. Imitation is a key skill in early social and academic learning from infancy [78] and is consistently reported as impaired in ASD [79]. In order to imitate, the child must foremost pay attention to others’ behaviors, which seems to be easier for children with emerging hyperlexic traits, perhaps related to the fact that they themselves direct more social communicative behaviors toward others, as discussed above. One can hypothesize that the increased directed social behaviors observed in children with hyperlexic traits might be related to the increased level of imitation also observed in these children.

The various emerging hyperlexic traits observed in the *ASD+eHPL* group and their developmental profiles can be contrasted with the elements of the hyperlexia definition. Indeed, *ASD+eHPL* children presented an ASD diagnosis along with an early interest and competence in letter and number recognition and naming that emerged around 3 years of age despite comprehension difficulties. Although no child in the *ASD+eHPL* group showed word reading, their letter and number skills remain special and early for their age. Nevertheless, our longitudinal analyses after one year showed no relationship between the *hyperlexic trait score* at baseline and change scores in autism symptoms or developmental and adaptive skills over the year. Thus, advanced letter and number skills were not related, either positively or negatively, to developmental changes over the year in our *ASD* sample. Indeed, despite the encouraging aspects highlighted in this study, this particular early skill may ultimately remain a mere passing interest and does not guarantee access to reading and writing later on [67]. However, because our sample of *ASD+eHPL* children was relatively small, it was not possible to draw definite conclusions regarding their developmental trajectory. Nonetheless, regardless of their subsequent developmental influence, the early hyperlexic traits represent an ultimately useful skill as letter and number knowledge is part of necessary school learning. Therefore, it is conceivable to take advantage of them in order to support children with ASD in their areas of weakness. Indeed, it has been suggested that children’s strengths should be supported in early intervention in autism, and not simply considered as reinforcers during social routines [80]. It is proposed that children with ASD could follow a specific developmental trajectory, in which language emerges later and which parents might nonetheless support. Instead of immediately and spontaneously engaging in oral language, children with ASD would allegedly first go through a stage of preferential processing of written stimuli such as subtitles or continuous scrolling of text, described as “autistic prerequisites” of oral language. As is often the case in ASD, the adaptation and individualization of educational strategies, in this case taking into account the early interest in written content, should not be neglected.

Our findings should be interpreted in light of their limitations. We used an ad hoc custom measure of early emerging literacy-related competencies to identify a small subsample of children with ASD who presented early hyperlexia-like traits. The sliding window nature of our measure limited its accuracy at the extremities of the age range that we studied. Indeed, despite the fact that the measure was extracted from a large sample of children aged 2 to 5 years old, the mean age of the first window used to identify the 90th percentile of the distribution was 2.50 years old, and the mean of the last window was 4.01 years old. This implies that, for children younger than 2.50 and older than 4.01 years old, we cannot defend with high certitude that the proposed cut-off of a *hyperlexic trait score* of, respectively, 4 and 6 is the most appropriate. However, we believe that this did not influence the identification of the *ASD+eHPL* group in the present study, given that in our sample, no children older than 4.01 years old had a score higher than 6, and that only eight children younger than 2.50 years old had a score higher than 0 (scores of 1 or 2). As such, we conclude that the measure that we propose can reliably be used in children aged 2.50 to 4.01 years of age, and that future studies should investigate whether it is appropriate for younger and older children. Regarding the measures used, data collection was not always possible for all children, for instance due to the late introduction of some assessments in our protocol, as was the case for the MSEL, changes caused by revised versions of the tests, such as the revision of the ADOS-G to the ADOS-2, validity issues or the inability to administer the complete test. In particular, the Written Communication subscale (VABS-II) was not always administered, as it is only normed from age 3 onwards. Therefore, its raw scores were not available for all children under the age of 3. In order to have a more accurate reading of the skills of children under 3 years of age on this subscale, it would have been of interest to have the raw scores of the entire sample. Finally, despite the fact that we used a fairly large sample of preschoolers, our results confirm that early hyperlexic traits are not a common feature of ASD (here, we report that 9% of children with ASD have early hyperlexic traits). As such, the resulting group of children with *ASD+eHPL* had a limited sample size, which might thus not be entirely representative of all children with early hyperlexic traits. Future studies with even larger sample sizes would be required to expand on the extensive characterization of these children’s developmental trajectories.

## 5. Conclusions

The current study quantified the early skills in naming and recognition of letters and numbers, which may represent a predisposition to subsequent hyperlexia in young preschoolers with ASD. Our results suggest that this early skill is associated not only with typical ASD language specificities such as stereotyped language or immediate echolalia but also with better expressive and written communication skills. Language comprehension, however, remains impacted, as is also the case in hyperlexia. Although the symptom profile of children with this particular skill is characterized by an increased level of RRB, more subtle behaviors of social interaction, such as vocalization and facial expression orientation, imagination and imitation, are better preserved. As these aspects may play an important role in early learning and pre-academic skills, early abilities in letters and numbers appear to be a positive skill that is promising for the future development of preschoolers with ASD.

## Figures and Tables

**Table 1:** Sample demographics at baseline.

		ASD (n = 155; 20♀)	TD (n = 30; 9♀)	*p*
Age		2.95 ± 0.72	2.87 ± 0.79	0.808
ADOS	SA	6.82 ± 2.18	1.03 ± 0.183	<0.001
	RRB	8.74 ± 1.60	2.13 ± 1.925	<0.001
	Total	7.66 ± 1.95	1.00 ± 0.00	<0.001
MSEL	Early learning composite	60.14 ± 22.74	110.89 ± 14.50	<0.001
VABS-II	Communication skills	74.45 ± 14.43	105.62 ± 12.07	<0.001
	Daily living skills	81.41 ± 13.11	102.48 ± 7.55	<0.001
	Socialization skills	77.41 ± 9.80	102.28 ± 7.55	<0.001
	Motor skills	86.98 ± 11.68	97.62 ± 7.70	<0.001

## Data Availability

As the collected data contain sensitive information of the families involved in the study, the data are not publicly available to avoid breach of patient confidentiality.

## Supplementary Materials

The following is available online at https://www.mdpi.com/article/10.3390/brainsci11060692/s1, Table S1: Group differences regarding the hyperlexic trait score, autism symptoms, developmental and adaptive measures.
