# Supplementary material for: Measuring the Emergence of Specific Abilities in Young Children with Autism Spectrum Disorders: The Example of Early Hyperlexic Traits
Source: Brain Sci. 2021 May 25;11(6):692. doi: 10.3390/brainsci11060692 (PMC8225194; doi:10.3390/brainsci11060692)
Supplement: Supplementary file 1 [file brainsci-11-00692-s001.zip › brainsci-1206465-SI.pdf]

Table S1. Group differences regarding the hyperlexic trait score, autism symptoms, developmental and adaptive measures.

		Group	*Median*	*IQR*	Pairwise Comparison	*U*	*z*	*p*	*r*
**Hyperlexic trait score**		ASD-eHPL	0	0	ASD-eHPL *vs.* ASD+eHPL	-88.175	-7.276	<.001***	-.533
		ASD+eHPL	6	2	ASD-eHPL *vs.* TD	-2.215	-.255	1.000	-.019
		TD	0	0	ASD+eHPL *vs.* TD	85.960	6.141	<.001***	.451
**ADOS**	SA	ASD-eHPL	7	3	ASD-eHPL *vs.* ASD+eHPL	828	-1.002	.316	-.080
		ASD+eHPL	5.500	4					
	RRB	ASD-eHPL	9	2	ASD-eHPL *vs.* ASD+eHPL	661	-2.182	.029*	-.175
		ASD+eHPL	10	1					
	Total	ASD-eHPL	8	4	ASD-eHPL *vs.* ASD+eHPL	941	-.292	.771	-.023
		ASD+eHPL	7.500	4					
**MSEL**	Visual Reception	ASD-eHPL	74.254	30.304	ASD-eHPL *vs.* ASD+eHPL	-11.086	-.912	1.000	-.006
		ASD+eHPL	71.982	41.074	ASD-eHPL *vs.* TD	-64.586	-6.576	<.001***	-.436
		TD	125.645	30.993	ASD+eHPL *vs.* TD	-53.500	-3.682	.001**	.025
	Fine Motor	ASD-eHPL	70.246	23.967	ASD-eHPL *vs.* ASD+eHPL	-15.911	-1.309	.572	-.108
		ASD+eHPL	77.118	22.086	ASD-eHPL *vs.* TD	-64.663	-6.584	<.001***	-.541
		TD	99.185	13.978	ASD+eHPL *vs.* TD	-48.752	-3.355	.002**	.276
	Receptive Language	ASD-eHPL	42.611	45.318	ASD-eHPL *vs.* ASD+eHPL	-22.861	-1.892	.175	-.013
		ASD+eHPL	55.592	54.211	ASD-eHPL *vs.* TD	-70.566	-7.228	<.001***	-.049

		TD	117.535	21.957	ASD+eHPL *vs.* TD	-47.705	-3.305	.003**	-.022
	Expressive Language	ASD-eHPL	42.322	29.928	ASD-eHPL *vs.* ASD+eHPL	-42.848	-3.548	.001**	-.292
		ASD+eHPL	64.868	32.124	ASD-eHPL *vs.* TD	-72.900	-7.336	<.001***	-.603
		TD	98.442	31.825	ASD+eHPL *vs.* TD	-30.052	-2.064	.117	-.170
**PEP-3**	Visual-Motor Imitation	ASD-eHPL	9	10	ASD-eHPL *vs.* ASD+eHPL	-19.864	-1.326	.554	-.097
		ASD+eHPL	11	9	ASD-eHPL *vs.* TD	-68.076	-6.334	<.001***	-.466
		TD	17	3	ASD+eHPL *vs.* TD	-48.212	-2.786	.016*	-.205
**VABS-II**	Communication skills	ASD-eHPL	71	17	ASD-eHPL *vs.* ASD+eHPL	-31.418	-2.069	.116	-.059
		ASD+eHPL	79	14	ASD-eHPL *vs.* TD	-83.106	-7.777	<.001***	-.578
		TD	110	19	ASD+eHPL *vs.* TD	-51.688	-2.958	.009**	-.220
	Receptive Communication	ASD-eHPL	9	4	ASD-eHPL *vs.* ASD+eHPL	-15.208	-1.006	.944	-.075
		ASD+eHPL	11	5	ASD-eHPL *vs.* TD	80.899	-7.601	<.001***	-.565
		TD	17	3	ASD+eHPL *vs.* TD	-65.691	-3.775	<.001***	-.281
	Expressive Communication	ASD-eHPL	9	3	ASD-eHPL *vs.* ASD+eHPL	-34.561	-2.288	.066	-.170
		ASD+eHPL	11	2	ASD-eHPL *vs.* TD	-89.309	-8.401	<.001***	-.624
		TD	17	3	ASD+eHPL *vs.* TD	-54.748	-3.150	.005	-.234

Written Communication	ASD-eHPL	3	6	ASD-eHPL *vs.* ASD+eHPL	-23.288	-2.822	.014*	-.031
	ASD+eHPL	6	3	ASD-eHPL *vs.* TD	-7.638	-.926	1.000	-.100
	TD	3	10	ASD+eHPL *vs.* TD	15.650	1.441	.449	.156
Daily Living skills	ASD-eHPL	82	20	ASD-eHPL *vs.* ASD+eHPL	-.240	-.016	1.000	-.001
	ASD+eHPL	85	17	ASD-eHPL *vs.* TD	-75.328	-7.048	<.001***	-.524
	TD	104	15	ASD+eHPL *vs.* TD	-75.088	-4.297	<.001***	-.319
Socialization skills	ASD-eHPL	76	11	ASD-eHPL *vs.* ASD+eHPL	-6.897	-.454	1.000	-.034
	ASD+eHPL	78	14	ASD-eHPL *vs.* TD	-87.004	-8.143	<.001***	-.605
	TD	100	13	ASD+eHPL *vs.* TD	-80.107	-4.586	<.001***	-.341
Motor skills	ASD-eHPL	85	17	ASD-eHPL *vs.* ASD+eHPL	-12.139	-.800	1.000	-.059
	ASD+eHPL	90	16	ASD-eHPL *vs.* TD	-52.078	-4.877	<.001***	-.363
	TD	96	8	ASD+eHPL *vs.* TD	-39.939	-2.288	.066	-.170

*p < .05; **p < .01; ***p < .001
